# Implementing a stigma reduction intervention in healthcare settings

**DOI:** 10.7448/IAS.16.3.18710

**Published:** 2013-11-13

**Authors:** Li Li, Chunqing Lin, Jihui Guan, Zunyou Wu

**Affiliations:** 1 Semel Institute for Neuroscience and Human Behavior, Center for Community Health, University of California at Los Angeles Los Angeles, CA, USA; 2 Fujian Provincial Center for AIDS/STD Control and Prevention, Fuzhou, China; 3 National Center for AIDS/STD Control and Prevention, Chinese Center for Disease Control and Prevention, Beijing, China

**Keywords:** HIV-related stigma, implementation, China, intervention

## Abstract

**Introduction:**

Globally, HIV-related stigma is prevalent in healthcare settings and is a major barrier to HIV prevention and treatment adherence. Some intervention studies have showed encouraging outcomes, but a gap continues to exist between what is known and what is actually delivered in medical settings to reduce HIV-related stigma.

**Methods:**

This article describes the process of implementing a stigma reduction intervention trial that involved 1760 service providers in 40 hospitals in China. Guided by Diffusion of Innovation theory, the intervention identified and trained about 15–20% providers as popular opinion leaders (POLs) to disseminate stigma reduction messages in each intervention hospital. The intervention also engaged governmental support in the provision of universal precaution supplies to all participating hospitals in the trial. The frequency of message diffusion and reception, perceived improvement in universal precaution practices and reduction in the level of stigma in hospitals were measured at 6- and 12-month follow-up assessments.

**Results:**

Within the intervention hospitals, POL providers reported more frequent discussions with their co-workers regarding universal precaution principles, equal treatment of patients, provider-patient relationships and reducing HIV-related stigma. Service providers in the intervention hospitals reported more desirable intervention outcomes than providers in the control hospitals. Our evaluation revealed that the POL model is compatible with the target population, and that the unique intervention entry point of enhancing universal precaution and occupational safety was the key to improved acceptance by service providers. The involvement of health authorities in supporting occupational safety was an important element for sustainability.

**Conclusions:**

This report focuses on explaining the elements of our intervention rather than its outcomes. Lessons learned from the intervention implementation will enrich the development of future programs that integrate this or other intervention models into routine medical practice, with the aim of reducing HIV-related stigma and improving HIV testing, treatment and care in medical settings.

## Introduction

Several decades into the HIV pandemic, HIV-related stigma continues to be a major challenge to prevention and treatment efforts worldwide [1–5]. Stigma in the general population has been well-documented, but its impact is also felt in healthcare settings [6, 7], where it can lead to testing avoidance, barriers to health counselling and a lack of adherence to antiretroviral therapies [8–10]. There is an urgent need for intervention efforts focused on reducing HIV-related stigma and discrimination, especially among frontline health service providers.

Globally, there has been substantial research on HIV-related stigma in healthcare settings. Previous studies have identified factors associated with stigma among service providers, including a lack of knowledge, fear related to the incurability of AIDS and prejudice toward marginalized behaviours [11, 12]. Our previous work identified a lack of institutional support and self-protection supplies as major reasons for avoiding service for people living with HIV in China [13, 14]. In 2009, Nyblade and colleagues conducted a literature review that identified strategies to combat stigma in healthcare facilities; their recommendations included using a participatory method, involving people living with HIV and training service providers on universal precautions [3]. Some intervention programs and activities tested in small-scale studies have shown encouraging outcomes [1, 15, 16]. For example, an intervention combining AIDS knowledge dissemination and contact with people living with HIV among 102 nursing students showed enhanced emotional competence to serve people living with HIV [16]. Our study team also conducted an intervention pilot in 2006 among 138 providers from four county hospitals in Yunnan, China. During the intervention delivery, people living with HIV acted as intervention trainers to share their experiences and facilitate discussions. Preliminary findings showed that provider participants in the intervention group reported better protection of patients’ confidentiality and lower levels of negative feelings toward people living with HIV [14]. However, one of the most critical issues impeding stigma reduction today is the gap between what is known about stigma and the systematic utilization of the evidence in full-scale intervention efforts in healthcare settings [17].

In light of our exploratory study findings and the promising outcomes of the pilot work [13, 14, 18], we implemented a randomized intervention trial that involved 1760 service providers in 40 hospitals in two provinces of China, with the objective to reduce service providers’ stigmatizing attitudes and behaviours towards people living with HIV in healthcare settings. The intervention efficacy was reported in another article published by the research team [19]. Rather than present the intervention outcomes, this article focuses on describing the implementation procedures in detail, reporting operational outcomes and sharing lessons learned.

## Methods

### Intervention framework

The intervention was designed using Diffusion of Innovation theory [20]. Instead of training every service provider in the hospitals, we identified, recruited and trained a subset of providers as popular opinion leaders (POLs) to communicate intervention messages to peers during everyday conversation and worked with them to sustain their advocacy activities [21]. The POL model has successfully been used to improve the quality of care by service providers in the United States [22, 23]. Our previous studies showed that the POL model is also applicable for service providers in China, given they are a stable population with an established network, and that some active and respected providers could potentially be effective change agents in stigma reduction within their professional community [18].

### Site selection and randomization

The study was conducted in Fujian and Yunnan provinces, China, from October 2008 to February 2010. These two provinces were selected because they represent the varied HIV rates and infection routes seen in the country [24, 25]. County hospitals were included because they are public facilities and easily accessed by most Chinese residents, and they are where many HIV infection cases are first detected. A total of 40 county hospitals were randomly selected from the 214 eligible hospitals in the two provinces.

The 20 hospitals from each province were first matched as pairs under comparable conditions such as number of beds, size of service provider staff, medical services offered and number of patients with HIV infection. After the baseline assessment, the two hospitals in each pair underwent a randomization process to assign them to an intervention group or control group. The geographic distance between the intervention and control hospitals was considered to avoid potential contamination.

### Identification of potential POL providers

To reach the goal of social norm shifting within the hospital, we targeted approximately 20 to 25 POLs from each intervention hospital, which covered about 15–20% of all providers [21, 26]. POL service providers in this study were deemed trustworthy, influential and reliable by their coworkers. Most importantly, the POL providers had to express care for their hospital and be willing to make an effort to improve the service quality of the facility. Three strategies were used to identify the POLs: 1) recommendations from department heads in the hospitals; 2) recommendations from co-workers; and 3) observations of the study's research staff.

The process was carried out as follows: department heads and other hospital administrators nominated persons they knew to be socially influential; then, the randomly selected providers who participated in the baseline assessment were asked to nominate the three most popular and influential providers in their hospital; and finally, our research staff observed the potential candidates’ interactions with their coworkers in order to verify the popularity of nominees and the strength of their social networks. To maintain balance and wide coverage, POLs were chosen from multiple departments in order to achieve broad coverage within each facility. The POL providers were recruited in two waves from each hospital, with about 10 to 13 POLs in each wave. The POLs in the first wave also participated in nominating POLs in the second wave.

### Recruitment of POL providers

The project recruiters approached potential POL providers after they were identified. Recruiters introduced the intervention as an opportunity to improve the POLs’ medical community, emphasizing that they were selected because of their influence and trustworthiness among colleagues. POLs were informed of their ethical rights, counselled on voluntary informed consent and invited to attend four weekly training sessions and bi-monthly reunion sessions. The refusal rate for POLs was less than 3%.

### Training of POL providers

Intervention facilitation teams were formed in both Fujian and Yunnan. The team consisted of local health educators, AIDS specialists and project staff. Prior to the intervention, all facilitators were given thorough training regarding institutional review board procedures, facilitator roles and responsibilities, intervention skills and protocol for emergency situations.

The selected POL providers attended four weekly group training sessions over a one-month period. Each session lasted about 1.5 hours and was held in a conference room at the county hospital where the providers worked. The participants were seated in a circle so that the facilitators could make eye contact with every person in the group. The titles of the four sessions were: 1) Complying with Universal Precaution Procedures and Ensuring Occupational Safety; 2) Fighting Against Stigma and Improving the Provider-Patient Relationship; 3) Taking Actions and Making Efforts to Care for Patients; and 4) Overcoming Difficulties and Building Up a Better Medical Environment. The intervention incorporated engaging activities such as discussion, games and role playing to encourage the trainees’ full participation. For example, a game called “Rescue Mission” conveyed the message of equal medical treatment of everyone regardless of their social status, type of disease, or infection route; and in a group discussion, “Discrimination Around Us,” providers were asked to identify discriminatory language and behaviours, especially in medical settings. Local elements were incorporated into the intervention materials. For example, local HIV-positive advocates and AIDS specialist stories were made into videos and demonstrated in the intervention sessions as real-life examples. The POLs received a small gift for each session of training activities they attended. The local teams selected inexpensive items, such as a pen or key chain, as a token of appreciation for their time and participation. To ensure the fidelity of intervention delivery, project evaluators observed every intervention session, assessed the quality of the intervention with a checklist and provided suggestions for improvement after each session.

### Dissemination of intervention messages from POL to peer providers

POL providers were encouraged to deliver intervention messages to their co-workers. The messages revolved around universal precautions and occupational safety, equal treatment of all patients, improvement of the provider-patient relationship and reduction of HIV-related stigma. To ensure broad message diffusion, POL providers were encouraged to talk to their coworkers not only within the same department, but also from other departments. Interactive techniques such as facilitator demonstration, group discussion, pair sharing and role playing were used to refine each POL's communication skills so that they could comfortably deliver messages. At the end of each intervention session, the POLs set goals to engage in conversations with coworkers, and the conversational outcomes were reviewed and discussed at subsequent sessions.

To ensure the sustainability of message dissemination, three reunion sessions were conducted after completion of the four initial training sessions. The first reunion was conducted one month after the initial training, while the second and the third reunions occurred four months after the previous reunion. The reunion sessions focused on group sharing, continued problem solving and skill building. For message delivery, the POLs reported in detail who they communicated with, under what circumstances, the contents of the conversation, challenges encountered and possible solutions. A group discussion about ways to improve the message delivery followed each POL's report.

### Provision of universal precaution supplies

To make structural changes in accessibility to supplies for self-protection, both intervention and control hospitals received information packages on general safety in medical procedures and universal precaution supplies from the National Center for AIDS/STD Control and Prevention (NCAIDS), Chinese Center for Disease Control and Prevention (CCDC). A Universal Precaution Oversight Committee was organized in each hospital under the supervision of the Infection Control Department to manage the dissemination of the supplies. Two or three volunteers from each hospital were assigned the role of supply managers; the supplies were distributed to the departments based on necessity; and supply managers were expected to report a shortage of universal precaution supplies to the Oversight Committee when necessary.

### Evaluation

At baseline, 44 providers were randomly selected from a publicly available staff roster of each participating hospital (total sample size=1760). In order to be eligible for the study, potential participants had to be aged 18 or above and work as a service provider (i.e., doctor, nurse, or lab technician) who had regular contact with patients. The POLs who were trained were not necessarily included in the assessments. At the time of recruitment, research staff followed a standardized script to explain the purpose of the study, procedures, confidentiality, voluntary participation and potential risks and benefits. Written informed consent was obtained prior to the data collection and study activities. The refusal rate was as low as 3%, and the follow-up rate was higher than 99% in both the intervention and control hospitals. At each assessment, providers completed a self-administrated paper-and-pencil questionnaire in a private room, with a trained interviewer available to answer questions. The survey took an average of 30 to 45 minutes to complete. Participants received 50 yuan (U.S. $8.00) for each assessment. All study documents and procedures were approved by the Institutional Review Boards of the University of California, Los Angeles, and the NCAIDS, CCDC.

Background information such as age, gender, profession and prior experience in treating people living with HIV was collected. The providers in the intervention hospital reported the frequency of intervention message diffusion and reception during the past six months at the 6- and 12-month follow-up assessments. Providers in all hospitals (both intervention and control) reported their perceived change in terms of universal precaution compliance, equal treatment of patients, provider-patient relationship and reduction in HIV-related stigma.

### Data analysis

Data were analyzed using SAS System for Windows (Version 9.2). We descriptively reported the background characteristics of POL providers in the sample. The times of message dissemination and reception during the past six months were compared between POL and non-POL providers in the intervention hospitals using a *t*-test; the perceived improvement in the hospital was compared between the intervention and control groups with a Chi-square test.

## Results

### Characteristics of POL providers

A total of 456 POL providers were included in the sample, the majority of whom were women (69.3%). The average age of the POL providers was 37.2 years at baseline. About one-third (38.6%) of providers had obtained an undergraduate medical degree or above. Two-thirds of the POL providers had worked in the medical field for more than 10 years. Slightly less than half (44.4%) of the POL providers were doctors, and 44.08% were nurses. The POLs were distributed among several departments: surgery, internal medicine, obstetrics-gynaecology, laboratory, emergency, STD and dermatology, otolaryngology, infectious diseases and paediatrics. Approximately 60% of POLs had prior contact with people living with HIV (Table 1).

**Table 1:** Characteristics of POL providers at baseline

	Number (Total=456)	%
Age (Mean=37.16, SD=8.35)		
Equal to or less than 30 years	91	19.96
31 to 40 years	206	45.17
41 years and above	159	34.87
Female	316	69.30
Medical education		
Vocational high school or below	91	19.96
Associate medical degree	187	41.01
Undergraduate medical degree or above	176	38.60
Years of medical service (Mean=15.04, SD=8.63)		
Equal to or less than 10 years	147	32.24
11 to 20 years	185	40.57
21 years and above	124	27.19
Profession		
Doctor	216	47.37
Nurse	201	44.08
Others	39	8.55
Department		
Surgery	99	21.71
Internal medicine	87	19.08
Obstetrics-Gynaecology (OBGYN)	86	18.86
Laboratory	37	8.11
Emergency	32	7.02
STDs and dermatology	29	6.36
Otolaryngology	24	5.26
Infectious diseases	23	5.04
Paediatrics	11	2.41
Others	28	6.14
Previous contact with people living with HIV	274	60.09

### Message dissemination and reception in intervention hospitals

Within the intervention hospitals, the POL providers reported more frequent message diffusion than non-POLs. For POLs, the average time spent discussing universal precaution compliance during the past six months was 9.29 minutes at the 6-month assessment and 10.45 minutes at the 12-month assessment, respectively. Conversely, the number was only 4.58 minutes at the 6-month assessment and 5.54 minutes at the 12-month assessment for non-POLs (*p*<0.0001 for both assessments). POLs disseminated messages of reducing HIV-related stigma at more than double the rate of non-POLs (29.45% vs. 10.21% at 6-months; 32.75% vs. 13.51% at 12-months). The POLs also discussed equal treatment of all patients and how to improve the provider-patient relationship significantly more often than non-POLs, at both the 6- and 12-month follow-up assessments (*p*<0.0001). In general, the message diffusion was more frequent at the 12-month than the 6-month assessment (Table 2).

**Table 2:** Message dissemination among intervention hospital providers

	6-Month follow up			12-Month follow up		
		
	Non-POL	POL		Non-POL	POL	
				
	N (%)	N (%)	p*	N (%)	N (%)	p*
In the past six months, how many times have *you talked to other providers in hospital* about …						
1. Universal precaution and occupational safety						
Mean±SD	4.58±7.28	9.29±9.65	<.0001	5.54±5.96	10.45±13.06	<.0001
0–2 times	182 (43.23)	77 (16.92)		142 (33.65)	64 (14.07)	
3–9 times	185 (43.94)	208 (45.71)		204 (48.34)	206 (45.27)	
10 times and above	54 (12.83)	170 (37.36)		76 (18.01)	185 (40.66)	
2. Equal treatment to all patients						
Mean±SD	4.13±7.37	8.27±9.54	<.0001	5.22±6.78	9.48±12.65	<.0001
0–2 times	217 (51.67)	94 (20.66)		165 (39.10)	95 (20.88)	
3–9 times	161 (38.33)	214 (47.03)		194 (45.97)	201 (44.18)	
10 times and above	42 (10.00)	147 (32.31)		63 (14.93)	159 (34.95)	
3. Improving provider-patient relationship						
Mean±SD	6.00±9.49	9.07±9.57	<.0001	6.57±8.08	10.27±12.34	<.0001
0–2 times	161 (38.24)	80 (17.58)		117 (27.73)	79 (17.36)	
3–9 times	177 (42.04)	202 (44.40)		218 (51.66)	193 (42.42)	
10 times and above	83 (19.71)	173 (67.58)		87 (20.62)	183 (40.22)	
4. Reducing HIV-related stigma						
Mean±SD	3.76±6.77	7.55±8.36	<.0001	4.78±5.41	8.25±9.59	<.0001
0–2 times	229 (54.39)	112 (24.62)		170 (40.28)	108 (23.74)	
3–9 times	149 (35.39)	209 (45.93)		195 (46.21)	198 (43.52)	
10 times and above	43 (10.21)	134 (29.45)		57 (13.51)	149 (32.75)	

* Two sample *t*-test.

The POLs also reported more reception of intervention messages from other providers in the hospital. At the 12-month assessment, POLs reported that peer providers in their hospital had talked to them an average of 10.25 times about universal precaution and occupational safety, 8.94 times about equal treatment, 10.13 times about improving provider-patient relationships and 8.31 times about reducing HIV-related stigma; while for non-POLs the numbers were 6.40, 5.41, 7.18 and 5.24, respectively (*p*<0.0001) (Table 3). There was no significant difference in message dissemination or reception between male and female providers.

**Table 3:** Message reception among intervention hospital providers

	6-Month follow up			12-Month follow up		
		
	Non-POL	POL		Non-POL	POL	
				
	N (%)	N (%)	p*	N (%)	N (%)	p*
In the past six months, how many times have *other providers in hospital talked to you* about …						
1. Universal precaution and occupational safety						
Mean±SD	5.38±7.54	8.15±8.01	<.0001	6.40±7.06	10.25±11.70	<.0001
0–2 times	153 (36.34)	63 (13.85)		105 (24.88)	56 (12.31)	
3–9 times	189 (44.89)	228 (50.11)		228 (54.03)	200 (43.96)	
10 times and above	79 (18.76)	164 (36.04)		89 (21.09)	199 (43.74)	
						
2. Equal treatment to all patients						
Mean±SD	4.46±7.05	7.08±6.77	<.0001	5.41±5.74	8.94±10.97	<.0001
0–2 times	196 (46.56)	104 (22.86)		147 (34.83)	81 (17.80)	
3–9 times	166 (39.43)	221 (48.57)		197 (46.68)	221 (48.57)	
10 times and above	59 (14.01)	130 (18.57)		78 (18.48)	153 (33.63)	
3. Improving provider-patient relationship						
Mean±SD	6.59±10.00	8.81±9.19	0.0006	7.18±8.94	10.13±12.80	<.0001
0–2 times	147 (34.92)	86 (18.90)		103 (24.41)	66 (14.51)	
3–9 times	181 (42.99)	210 (46.15)		207 (49.05)	209 (45.93)	
10 times and above	93 (22.09)	159 (34.95)		112 (26.54)	180 (39.56)	
4. Reducing HIV-related stigma						
Mean±SD	3.89±6.64	6.94±8.04	<.0001	5.24±6.22	8.31±11.14	<.0001
0–2 times	226 (53.68)	112 (24.62)		156 (36.97)	111 (24.40)	
3–9 times	156 (37.05)	231 (50.77)		199 (47.16)	202 (44.40)	
10 times and above	39 (9.26)	112 (24.62)		67 (15.88)	142 (31.21)	

* Two sample *t*-test.

### Distribution of universal precaution supplies

During the 12-month follow-up period, each of the 40 participating hospitals received 100 disposable sharp containers, 50 disposable cloths, 50 disposable waterproof aprons, 15 pairs of protection goggles and 100 pairs of rubber gloves. The amount of supply distribution was the same for the intervention and control hospitals. For the hospitals, this was the first time to see a gesture from the government to promote universal precaution practice.

### Perceived improvement in the hospitals

Compared to the control group, the intervention hospital providers perceived more improvement in universal precaution and occupational safety, equal treatment of all patients, provider-patient relationship and reduction in HIV-related stigma. For example, more than half (55.19%) of the providers in the intervention hospitals reported significant improvement in universal precaution and occupational safety in their hospitals at the 12-month assessment, while only 28.18% of the control hospital providers felt that way. The proportion of the intervention providers who perceived a significant reduction in HIV-related stigma at the 12-month assessment was more than double the number among the control providers (45.50 vs. 20.68%). The perceived improvement was sustained and augmented at 12 months (Table 4).

**Table 4:** Perception of improvement in the hospital

	6-Month follow up			12-Month follow up		
		
	Control	Intervention		Control	Intervention	
				
	N (%)	N (%)	p*	N (%)	N (%)	p*
In the past six months, do you think *your hospital has improvement* in terms of …						
1. Universal precaution and occupational safety						
Significantly	241 (27.48)	376 (42.92)	<.0001	248 (28.18)	484 (55.19)	<.0001
Some	547 (63.37)	463 (52.85)		557 (63.30)	368 (41.96)	
No improvement	51 (5.82)	20 (2.28)		45 (5.11)	10 (1.14)	
No judgment	38 (4.33)	17 (1.94)		30 (3.41)	15 (1.71)	
2. Equal treatment to all patients						
Significantly	216 (24.63)	307 (35.05)	<.0001	224 (25.45)	405 (46.18)	<.0001
Some	538 (61.35)	501 (57.19)		562 (63.86)	434 (49.49)	
No improvement	65 (7.41)	25 (2.85)		57 (6.48)	12 (1.37)	
No judgment	58 (6.61)	43 (4.91)		37 (4.20)	26 (2.96)	
3. Provider-patient relationship						
Significantly	366 (41.73)	394 (44.98)	0.2994	364 (41.36)	489 (55.76)	<.0001
Some	449 (51.20)	431 (49.20)		459 (52.16)	355 (40.48)	
No improvement	38 (4.33)	26 (2.97)		38 (4.32)	19 (2.17)	
No judgment	24 (2.74)	25 (2.85)		19 (2.16)	14 (1.60)	
4. Reducing HIV-related stigma						
Significantly	188 (21.44)	273 (31.16)	<.0001	182 (20.68)	399 (45.50)	<.0001
Some	477 (54.39)	488 (55.71)		513 (58.30)	410 (46.75)	
No improvement	109 (12.43)	43 (4.91)		103 (11.70)	24 (2.74)	
No judgment	103 (11.74)	72 (8.22)		82 (9.32)	44 (5.02)	

* Chi-square test.

## Discussion

This article describes the process of implementing a large-scale stigma reduction intervention trial in general health settings in China. This study has limitations. For example, the frequency of message diffusion relied on self-reports, making social-desirability bias a concern. Also, as the POLs took part in the intervention, they might be more sensitive to the intervention messages and tend to report more frequent message dissemination than non-POLs. Additionally, we were not able to measure the real usage of universal precaution supplies in the facilities. In spite of these limitations, we learned a number of lessons in the course of implementing the project.

There were some difficulties we encountered during the POL training. First, the provider participants all had busy work schedules. To ensure that all POLs could participate, the field staff communicated with the POL participants beforehand to seek their opinion on the preferred time for conducting sessions. The sessions were usually conducted in late afternoons after work or during midday breaks. Second, some providers were not used to the interactive format and reluctant to talk at the beginning, so the facilitators re-emphasized that there was no right or wrong answer, and encouraged the participants by giving positive reinforcement and recognition throughout the sessions to prompt optimum sharing. Third, some POL providers insisted that no stigma exists in their facility, or that people living with HIV deserved to be discriminated against because of their “immoral” behaviours. In these cases, the facilitators still showed respect for the participants and used games and group discussion to address their attitudes.

The reunion sessions proved to be an important platform to share experiences and skill building among POL providers, and also served as a source for feedback collection for the researchers. During reunion sessions, the POL providers reported that they conveyed stigma reduction message not only in words but in their personal actions, and the messages were generally well-accepted by their audience. Some POL providers encountered peers who perceived HIV to be far-removed from their lives and the topic was irrelevant, especially in areas with low HIV prevalence, but their perception and awareness of the issue could be changed through repeated conversation.

One lesson we learned from this study was to find a unique entry point when implementing the intervention. It is genuinely challenging to engage service providers in a stigma reduction intervention because they are regarded as experts in the medical field. Instead of solely disseminating knowledge and identifying stigmatizing attitudes and behaviours, the intervention addressed occupational safety concerns by promoting universal precaution as a way of self-protection at work. This strategy built upon our previous studies that discovered a lack of universal precaution knowledge and supplies among providers, and its relationship with the providers’ avoidance attitudes to serve people living with HIV [27, 28]. This approach was well accepted by the participating providers, and we received feedback from participants that the intervention message was very relevant to their self-interests. By adhering to universal precautions in their medical practice, the service providers released fear of occupational exposure and became more willing to serve HIV-positive patients.

During the implementation of an intervention, it is important to recognize its community context and use culturally appropriate intervention strategies [29–31]. For this project, we focused on preserving the fidelity of the intervention component while also incorporating local elements. The involvement of experienced local educators and use of local language enhanced the acceptability and sustainability of the intervention. In addition, we identified HIV specialists and local representatives of people living with HIV and presented their personal stories during the intervention sessions. Such real-life stories reminded service providers of the existence of stigma and inspired them to follow the community role model to make changes in their professional environment.

Governmental support in making changes at the structural level was crucial to the stigma reduction project [31]. From a previous study, we discovered that stigma among service providers was largely influenced by structural barriers such as the availability of universal precaution [13]. The intervention project successfully engaged NCAIDS, CCDC, the leading HIV/STD control agency in China, to allocate about 100,000 yuan (approximately U.S. $15,000) in subventions for universal precaution supplies to the participating hospitals. Although this funding was insufficient to meet the demand for universal precaution supplies in all hospitals, the action was regarded as a clear gesture of the involvement of health authorities in supporting occupational safety, which further initiated safer medical practice conversations among local hospital administrators. Following the action of NCAIDS, CCDC, hospitals in the intervention condition made further purchases of universal precaution supplies. The structural change was thus sustained beyond the project period and was translated into the service providers’ routine medical practice.

## Conclusions

This article describes the implementation process of an intervention program that has the potential to reduce HIV-related stigma in medical settings. Since the intervention focuses on equal treatment for all patients, it can easily be applied to stigma reduction programs in a number of different populations. During the adaptation, however, one should consider the participants’ needs and recognize culture and community contexts. Policy support in structural change is warranted to incorporate the intervention into existing healthcare settings to ensure sustained outcomes.
